# Pulmonary benign metastasizing leiomyoma: a report of two cases

**DOI:** 10.2144/fsoa-2022-0021

**Published:** 2022-09-23

**Authors:** Mohammad A AlQudah, Shadi Hamouri, Hala K Haddad, Ra'fat Tawalbeh, Husam K Haddad

**Affiliations:** 1 Department of Pathology & Microbiology, Faculty of Medicine, Jordan University of Science & Technology, Irbid, 21110, Jordan; 2 Department of Surgery & Urology, Faculty of medicine, Jordan University of Science & Technology, Irbid, 21110, Jordan; 3 Department of Ophthalmology, Royal Medical Services, Irbid, 21110, Jordan; 4 Department of Pathology & Laboratory Medicine, Ministry of Health, Amman, 11118, Jordan

**Keywords:** benign metastasizing leiomyoma, lung metastasis, myomectomy, pulmonary nodules

## Abstract

Benign metastasizing leiomyoma (BML) is a rare pathological process associated with pelvic leiomyoma. We present two cases of BML that are associated with giant pulmonary metastasis. BML is a rare benign metastatic phenomenon that could easily be mistaken for malignant neoplasms. Both cases occurred in middle-aged women who presented with cough and dyspnea. They previously underwent hysterectomy for uterine leiomyoma. After history taking, computed tomography, integrated PET/computed tomography and pathological assessment, a multidisciplinary treatment was offered for the diagnosis of BML. Physicians should consider BML among the differential diagnoses in women of reproductive age with a history of uterine leiomyoma presenting with pulmonary nodules, and accurate histopathological analysis should be made.

## Plain language summary

This article reports two cases of a rare pathological process called benign metastasizing leiomyoma. Since it can be mistaken with malignant neoplasm, the report provides evidence and guidance for clinicians and histopathologists to be considered when dealing with similar cases.

Benign metastasizing leiomyoma (BML) is a rare benign metastatic phenomenon that is related to pelvic leiomyoma. Histologically, the primary and metastatic lesions are characterized by benign appearing smooth muscles with histological, immunological and molecular patterns that mimic those related to benign uterine leiomyomas [1]. Upon histopathology, BML lesions are well-differentiated spindle cells usually showing certain features, including low mitotic rate (<5/10 high power field), slight cytological atypia and distinct cell borders [2]. The immunohistochemical staining shows positivity for smooth muscle actin, keratin and desmin immunostains in addition to the positivity for estrogen (ER) and progesterone receptors (PR), which indicated its uterine origin [3,4].

Steiner first described BML in 1939 [1]. He described that the associated extrauterine metastases prove to be malignant. It can metastasize to several organs, with the lung being the most common target organ. In relatively few cases, BML affected various organs, such as the skin, bladder, esophagus, liver, bone and even albeit rarely the spine [5,6].

Pulmonary BML usually presents with respiratory symptoms, most commonly shortness of breath, cough and more rarely hemoptysis, hemothorax or pneumothorax [5]. All BML cases are usually associated with a positive history of uterine surgery [3]. Given the disease’s indolent course, most patients will have evidence of pulmonary nodules on computed tomography (CT) scan at the time of presentation, followed by a tissue biopsy to confirm the diagnosis [7].

Herein, we describe two cases of BML that present with giant pulmonary lesions 5 and 17 years after hysterectomy for uterine fibroids.

## Case 1

**Figure 1.**
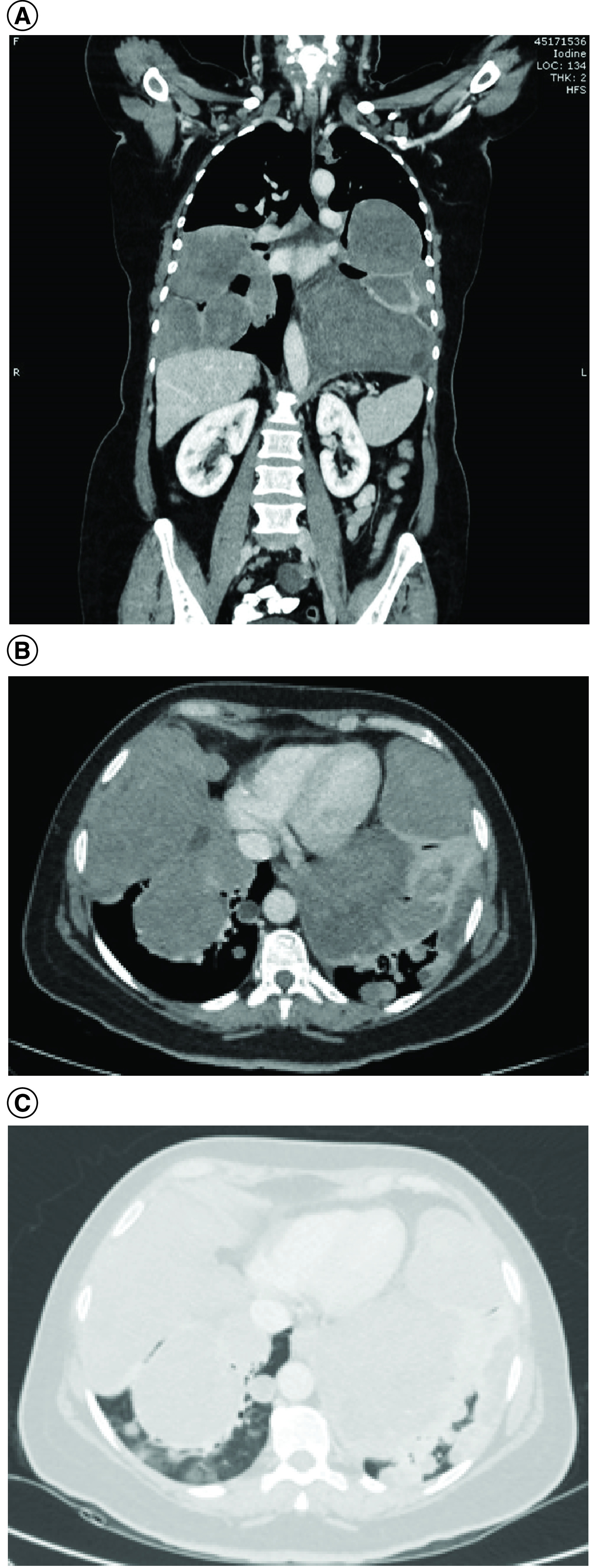
Computed tomography scan showing multiple enlarged bilateral pulmonary nodules. **(A)** Coronal soft tissue window. **(B)** Axial soft tissue window. **(C)** Axial lung window.

A 51-year-old women presented with cough for 1 year and shortness of breath for 4 months. The patient is para 3; all were normal vaginal deliveries at full term. She had a history of uterine fibroids and myomectomy 17 years ago. CT showed multiple enlarged bilateral pulmonary nodules, the largest measuring 11.7 × 11.2 × 11 cm (Figures 1A–C). Abdominopelvic CT showed heterogeneous soft tissue mass at the uterine cervix junction measuring 3.8 × 3.1 cm with a well-defined cystic lesion in the left adnexa measuring 3 cm.

**Figure 2.**
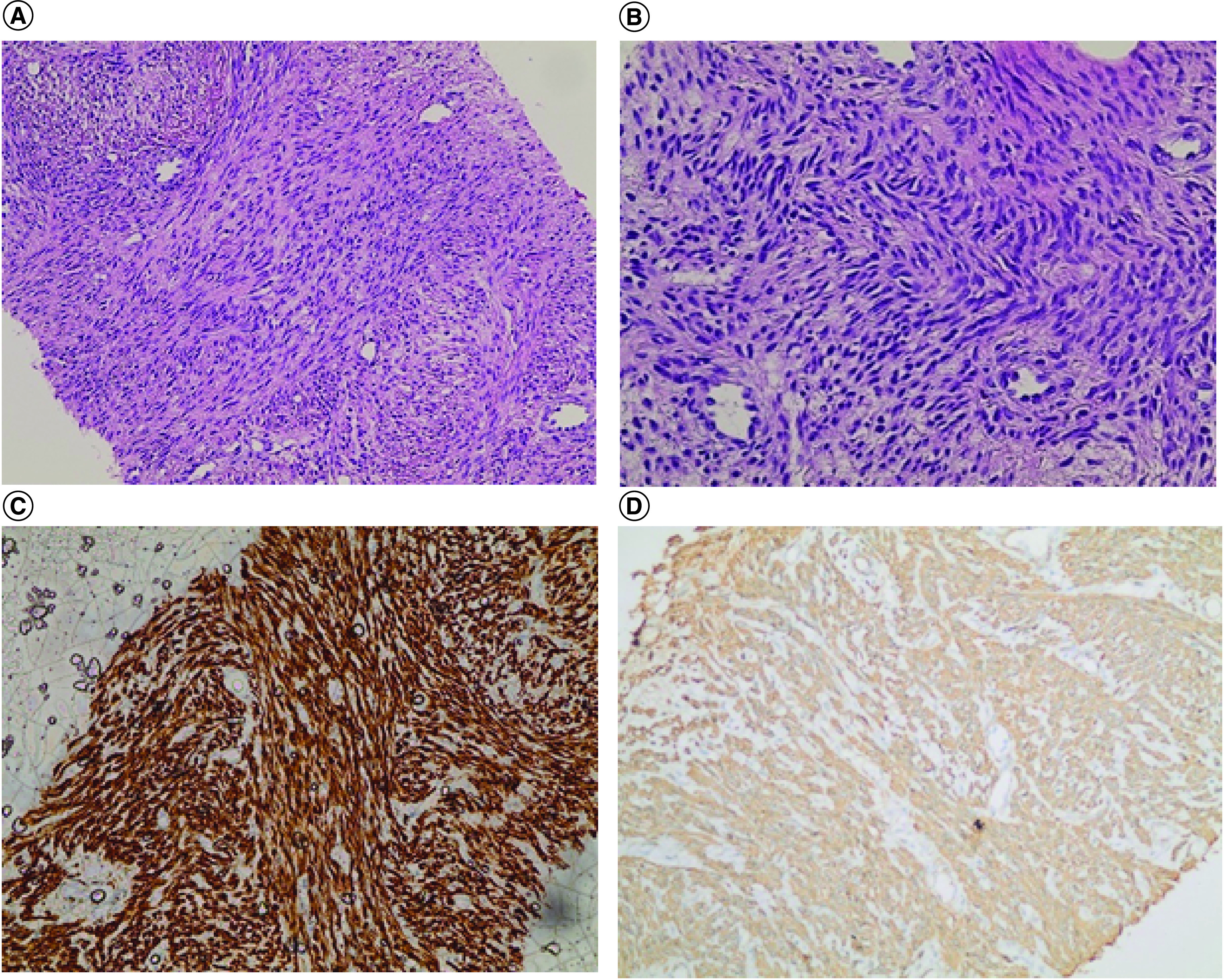
Spindle cell lesion with fascicular pattern and intersecting at right angles. The cells show cigar-shaped nuclei without necrosis, mitosis, and nuclear pleomorphism. The cells are immunopositive for desmin and smooth muscle actin immunostains. **(A)** Hematoxylin and eosin. **(B)** Hematoxylin and eosin. **(C)** Desmin. **(D)** Smooth muscle actin.

Brain CT was free. PET showed numerous large hypermetabolic masses, and the largest one was located in the left upper lobe, measuring 10.2 × 6.8 cm, with no evidence of abnormal focal uptake in the neck, chest or abdomen. CT-guided biopsy showed benign spindle-cell proliferation forming fascicles that intersect at right angles, which confirmed pulmonary BML. The tumor cells showed cigar-shaped nuclei with no evidence of tumor necrosis, mitosis or nuclear pleomorphism. The tumor cells were positive for smooth muscle actin, desmin, ER, PR and BCL2 immunohistochemical markers and negative for CD99, CD34, CD31, S-100 protein and beta-catenin immunohistochemical markers (Figures 2A–D).

The pulmonary function test showed severe restrictive lung disease with a forced vital capacity (L) of 48% and forced expiratory volume in the first second (L) of 46%. The patient was started on hormonal therapy, including letrozole, an aromatase inhibitor, goserlin and gonadotropin releasing hormone agonist and planned for total abdominal hysterectomy with bilateral salpingo-oophorectomy.

## Case 2

In 2013, a 47-year-old women presented to the emergency department at King Abdullah University Hospital with a cough and shortness of breath. She had a history of diffuse uterine leiomyomatosis, for which she underwent a hysterectomy in 2008. Chest CT showed bilateral multiple variably sized pulmonary masses and nodules. The results of a CT-guided biopsy appeared inadequate. Therefore, video-assisted thoracoscopic lung-wedge resection was performed, which confirmed pulmonary BML by showing a well-demarcated grayish nodule measuring 1.5 × 1.5 × 1 cm, composed of epithelioid cells arranged as nests and separated by steaks of hyalinized stroma. The cells showed no atypia, and the nuclei were vesicular with small nucleoli. Mitotic figures were up to 4/10 high power field with no apparent necrosis. The tumor cells were strongly positive for SMA, desmin and ER. The specimen appeared hostile for HMB-45, CD10, CK, S-100 protein, synaptophysin, chromogranin, CD34 and thyroid transcription factor 1, with Ki-67 showing 3% proliferative activity.

In 2014, she underwent bilateral salpingo-oophorectomy and pelvic lymph node biopsy. The pelvic lymph node biopsy showed similar histopathological features to the previously mentioned pulmonary nodule leading to the diagnosis of BML. Later, in 2015, the patient presented with sudden shortness of breath and chest pain. CT showed pulmonary embolism in the posterior right pulmonary vein with multiple bilateral pulmonary nodules that increased in size. Unfortunately, in the same year, the patient died of massive pulmonary embolism.

## Discussion

Pulmonary BML is thought to affect women at a mean age of 47.3 ± 10.02 years with a previous history of uterine leiomyoma and primary surgery at a mean age of 38.5 ± 8.99 years. The mean age at primary surgery correlated with the mean age of diagnosis [5]. Despite that, only a few cases were reported for cases with an intact uterus and no previous history of leiomyoma [5,8].

The exact pathogenesis of BML has not been completely identified yet. Various theories have been proposed to understand its pathogenesis better; peritoneal seeding after myomectomy or hysterectomy, benign smooth muscle cells metastasizing from uterine leiomyoma and low-grade uterine leiomyosarcoma. The peritoneal seeding hypothesis was reported based on that most of the patients previously underwent hysterectomy or myomectomy, suggesting a surgically related hematogenous spread and that BML and intravascular leiomyomatosis are related entities [4,9,10]. Despite that, in some cases, the uterine tumor was discovered simultaneously or after the associated metastasis [11].

A multifocal origin has also been linked to BML in multiple reports. However, extrauterine leiomyomas are usually ER negative, whereas most BML is ER positive and heavily relies on ER and PR, with the disease occurring in premenopausal women and the tumor demonstrating growth reduction post menopause or during pregnancy [11]. Therefore, hormonal therapy controlling estrogen levels is deemed effective in preventing tumor progression with a good prognosis [7]. The third hypothesis relates BML to low-grade uterine leiomyosarcoma. Despite that, pulmonary BML is considered much less aggressive than leiomyosarcoma, with several asymptomatic cases found incidentally in routine radiological investigations [11].

In 2020, Kayser *et al.* reported that the mean interval between hysterectomy and the development of BML was 14.9 years, whereas in 2017, Barnaś *et al.* reported it as 8.8 years regardless of the surgery type. Of the two patients, one was diagnosed 5 years after hysterectomy, whereas the second was diagnosed 17 years after myomectomy.

The treatment for pulmonary BML has not been standardized yet. Among those is surgery by excising the foci and observing the development of new foci [7]. Other options work on its hormonal relation, whether through oophorectomy or medical castration, altering the hormone release and stabilizing the pulmonary lesion. Oophorectomy was previously linked to progressive and complete regression of some low-grade BML cases [12]. This modality of hormonal control was challenged by hormonal treatment as an effective, reversible and less aggressive alternative. Moreover, hormonal replacement is superior to oophorectomy in inoperable cases and women who desire to retain their fertility and can be used in progressing diseases despite oophorectomy or menopause [11,13].

## Conclusion

BML is a rare borderline tumor combining benign histological features with a metastasizing biological behavior indicating malignancy. It should be in the differential diagnoses of women of reproductive age with a history of uterine disease presenting with pulmonary nodules; thus, an accurate histopathological analysis should be performed. To the best of our knowledge, fewer than ten cases were reported in the Middle East, with no previous cases reported in Jordan.

**Executive summary**

- Benign metastasizing leiomyoma (BML) is a rarely reported phenomenon that primarily affects women with a previous history of uterine leiomyoma.
- Various theories are proposed to understand the pathogenesis of BML including peritoneal seeding after myomectomy, benign smooth muscle that metastasize from uterine leiomyoma and low-grade uterine leiomyosarcoma.
- Hormonal therapy can be an effective modality in preventing tumor progression.
- BML should be among the differential diagnoses in women of reproductive age with a history of uterine disease when presenting with pulmonary nodules.
